# Ocular Involvement in Pemphigus Vulgaris Without Skin Lesions: A Case Report

**DOI:** 10.7759/cureus.26309

**Published:** 2022-06-24

**Authors:** Iyad Majid, Brandon R Martel, Melanie Martel, Leslie K Tamura

**Affiliations:** 1 Ophthalmology, Martel Eye Medical Group, Rancho Cordova, USA; 2 Ophthalmology, California Northstate College of Medicine, Elk Grove, USA; 3 Ophthalmology, University of California Davis School of Medicine, Sacramento, USA; 4 Internal Medicine, Mercy General Hospital, Carmichael, USA

**Keywords:** atypical pemphigus vulgaris, drug-induced allergic response, dermatology, autoimmune blistering diseases, pemphigus vulgaris, ophthalmology

## Abstract

Pemphigus vulgaris (PV) is an autoimmune disorder affecting the skin and mucous membranes. The condition may be confused with a number of disorders, including Stevens-Johnson syndrome (SJS), toxic epidermal necrolysis (TEN), and erythema multiforme (EM), all of which are life-threatening. Immunohistological and histochemical analyses remain the optimal methods for differentiating these diseases. There is still insufficient evidence regarding the true incidence rate of ocular disease in PV as well as its distinct clinical types. This report sets to review the case of a 62-year-old male with atypical ocular pemphigus vulgaris and review the literature.

## Introduction

Pemphigus vulgaris (PV) is a rare autoimmune disorder characterized by the development of flaccid blisters and erosions (typically in the oral cavity) on otherwise normal skin or mucosal membranes. The immune hypersensitivity condition may be confused with a number of other disorders, including Stevens-Johnson syndrome (SJS), toxic epidermal necrolysis (TEN), and erythema multiforme (EM), all of which were previously believed to be the same disease [1].

PV is often caused by an unusual allergic reaction or immune response to various antibiotics or medications. PV may be life-threatening if not attended to; it leaves a patient vulnerable and susceptible to a number of potentially fatal complications including infections, and fluid and electrolyte disturbances. These issues can lead to severe sepsis and cardiopulmonary failure. A patient with autoimmune disease must be attended to as early as possible to prevent the further development of dangerous complications [2].

Although research demonstrates the oral cavity to be the most common mucosal site of involvement, the ocular surface may also be affected, with the first report of ocular involvement in PV in 1975 [3]. Since then, a number of case studies of PV patients with ocular complications have been described. As evident by these reports, ocular involvement in PV is infrequent, although it is likely underdiagnosed, where the symptom only shows up less than 17% of the time [2,4].

Information from the patient’s history, such as exposure to specific drugs or infections, and clinical information, such as the characteristics of skin blisters, have been useful in differentiating among PV, SJS, TEN, or EM. A biopsy taken at the edge of a blister or inflamed skin and immunohistological and histochemical analyses remain the optimal methods for differentiating these diseases; a simple biopsy alone only yields a suggestive diagnosis [5,6].

The little data regarding the real incidence rate of ocular manifestation in PV might be owing to a paucity of large-scale investigations concentrating on the matter. Furthermore, only individuals with severe ocular problems are included in previous case reports and case series. Ocular symptoms are likely to be modest and unobtrusive, so a complete ophthalmologic examination is generally necessary to discover minor changes [7,8]. This case gives insight into the diagnostic and treatment methods for the underdiagnosed atypical pemphigus vulgaris with ocular involvement and without skin lesions.

## Case presentation

A 62-year-old male with a history of coronary artery disease status post-four-vessel coronary artery bypass grafting and insulin-dependent diabetes mellitus type 2 was seen by his primary care doctor for progressive mucositis with odynophagia and dysuria. His symptoms included blood and sores on his lips; swollen lips; extreme pain when swallowing, talking, and urinating; irritation around and over the eyes; and sensitivity to light. Soon after, the patient began to develop conjunctivitis. The conditions were secondary to an unknown etiology.

A few weeks earlier, the patient had been diagnosed with *Helicobacter pylori* (*H. pylori*) gastritis on esophagogastroduodenoscopy (EGD), for which he was prescribed amoxicillin (which he has had before), clarithromycin, and pantoprazole for 14 days. After taking these medications, he developed mucositis on his lips first and then on his penile and rectal areas. He also began experiencing erythema of conjunctiva accompanied by tearing.

The patient was ultimately presented to the hospital for inpatient evaluation after trying other outpatient treatments: viscous lidocaine, magic mouthwash, nystatin swish and swallow, and diflucan. During his initial hospital workup, he was treated for possible atypical viral or fungal infection. Infectious disease service was contacted, and he was prescribed antibiotics, antifungals, and antiviral medications and was ordered other serologies. The patient’s condition did not improve; rather, it worsened.

A blood serology was performed to measure the levels of antinuclear antibodies (ANA). As depicted in Table 1, the test detected that the patient was ANA-positive, denoting an autoimmune disorder. The titers were twice as high as the upper limit of the reference range. He also had increased levels of *Chlamydia pneumoniae* immunoglobulin G (IgG) titer, implying an immune response to a prior exposure to the organism. Toxicology through urine reported negative for amphetamines, barbiturates, benzodiazepines, cocaine, methadone, opiates, PCP screen, THC, and oxycodone.

**Table 1:** Blood serology test of the 62-year-old patient with pemphigus vulgaris.

Laboratory parameters	Reference ranges	Patient values
ANA HEp-2 IgG (IU/mL)	1:19-1:80	1:160
Antinuclear AB (IU/mL)	1:19-1:80	1:160
IgG (IU/mL)	1:19-1:80	1:160
ANA titer (IU/mL)	1:19-1:80	1:160
ANCA IFA titer (IU/mL)	1:19-1:80	<1:20
*Chlamydia pneumoniae* IgG titer (IU/mL)	1:64	1:128
*Mycoplasma pneumoniae* IgM		Negative
Herpes simplex virus (HSV)		Negative
Enterovirus		Not detected
Treponemal antibodies		Nonreactive

During his hospitalization, the patient tolerated an EGD with biopsy (which revealed mucositis) and two lip biopsies by an otorhinolaryngologist (ENT). Due to no cutaneous involvement, an outpatient dermatologist and a plastic surgeon at another facility determined SJS and TEN to be unlikely the cause of symptoms. The dermatologist evaluated the patient via telemedicine and determined that he had a “blistering disease” with paraneoplastic pemphigus or an autoimmune disease; the doctor then recommended high-dose steroids to combat pervasive issues. Although there were no blisters on the skin, the skin on the patient’s elbows and abdomen began to break down.

After careful review, it was deduced that these symptoms, including the initially diagnosed mucositis, were related to a drug-induced allergic response. The lip histopathology findings were suggestive of pemphigus vulgaris. The biopsy showed spongiosis in the lower spinous cell layers, as well as suprabasal epithelial clefts. Lymphocytes and neutrophils were detected to be the main cells causing an inflammatory response in the clefts. The biopsy also demonstrated acantholysis in the lower spinous cell layers with detached keratinocytes. To confirm these findings, direct immunofluorescence testing on freshly biopsied tissue was needed; the technique gave a definitive diagnosis, as it highlighted immunoglobulin G (IgG) antibodies in the lip spinous cell junctions. The positive level of IgG in the patient confirms these findings. Following this finding, previous medications were ceased, and steroid medication was administered to aid in the healing process.

## Discussion

Pemphigus vulgaris is recognized by “flaccid, mucous, or mucocutaneous blisters” [9]. With most affected individuals, the oral mucosa is primarily affected (with other mucous membranes sometimes involved as well). Typically, skin lesions form at any location of the tegument [10]. There appears to be, however, rare and atypical instances where skin lesions do not appear on the patient, which may render a delayed or incorrect diagnosis.

The ocular symptoms the patient experienced were burning and irritated feeling around and over the eye, foreign body sensation in the eye, sensitivity to light, and dry eyes. At first, the absence of skin lesions puzzled doctors, but after the suggestive lip biopsy and the confirming direct immunofluorescence, the patient was diagnosed with and soon treated for pemphigus vulgaris.

There is controversy in the management of atypical ocular PV as it can be self-limiting. Treatment with antibiotics, antifungals, antivirals, and/or other drugs and medications may cause the disorder to worsen. As evident in the case report through the patient’s endoscopy, the autoimmune disease results from a drug-induced response; the addition of drugs or medications may yield the production of more antinuclear antibodies, which would lead to a secondary worsening of mucosal membranes [11]. Thus, different treatment methods must be used to ensure the patient’s safety and full recovery.

First, immunohistology or immunofluorescence testing is required to make a definitive diagnosis. Simple biopsies only yield suggestive analyses for diagnosis; they may demonstrate acantholysis, but IgG antibodies are shown only by direct immunofluorescence. Pathology of the conjunctiva was not obtained as the first specimen identified the problem. Sample tissue should be taken at the edge of an affected lesion to include mucosal tissue just outside the lesion to comprise adequate non-blistered and non-ulcerated tissue for proper evaluation. In addition, indirect serologic testing could also be considered. Then, the same course of treatment used for similar diseases (i.e., SJS, TEN, and EM) is done to combat PV: the stopping of medications for five days and the use of steroids and immunosuppressive agents [1]. Antibiotics may be carefully administered after the dead period to combat any ongoing infections, although the patient must be attentively observed to ensure that the disease does not worsen.

## Conclusions

Pemphigus vulgaris may be life-threatening if not addressed and treated. A patient diagnosed with PV will likely be identified by issues spurred by infectious diseases and should seek treatment immediately. The absence of skin lesions, paired with the infrequent involvement of the eyes, may lead to similar atypical cases of PV being unnoticed and undiagnosed. However, this report presented symptoms and diagnostic methods for this rare autoimmune disorder.

To begin treatment, a simple biopsy must first be taken at the edge of a blister or inflamed skin and should be paired with immunohistology or immunofluorescence testing to make a definitive diagnosis. Then, the patient should stop receiving medications for five days. After the dead period, a patient may be administered steroids to aid in recovery.

## References

[1] Anders UM, Taylor EJ, Kravchuk V, Martel JR, Martel JB (2015). Stevens-Johnson syndrome without skin lesions: a rare and clinically challenging disease in the urgent setting. Emerg Med Open J.

[2] Gregoriou S, Efthymiou O, Stefanaki C, Rigopoulos D (2015). Management of pemphigus vulgaris: challenges and solutions. Clin Cosmet Investig Dermatol.

[3] Bean SF, Halubar K, Gillett RB (1975). Pemphigus involving the eyes. Arch Dermatol.

[4] Daoud YJ, Cervantes R, Foster CS, Ahmed AR (2005). Ocular pemphigus. J Am Acad Dermatol.

[5] Kuriachan D, Suresh R, Janardhanan M, Savithri V (2015). Oral lesions: the clue to diagnosis of pemphigus vulgaris. Case Rep Dent.

[6] Suliman NM, Åstrøm AN, Ali RW, Salman H, Johannessen AC (2013). Clinical and histological characterization of oral pemphigus lesions in patients with skin diseases: a cross sectional study from Sudan. BMC Oral Health.

[7] Laforest C, Huilgol SC, Casson R, Selva D, Leibovitch I (2005). Autoimmune bullous diseases: ocular manifestations and management. Drugs.

[8] Camisa C, Meisler DM (1992). Immunobullous diseases with ocular involvement. Dermatol Clin.

[9] Tariq U, Nasrullah A, Guha A, Mitre M (2021). Oroesophageal pemphigus vulgaris secondary to lisinopril use: a new side effect. Cureus.

[10] Mihai S, Sitaru C (2007). Immunopathology and molecular diagnosis of autoimmune bullous diseases. J Cell Mol Med.

[11] Pile HD, Yarrarapu SNS, Crane JS (2022). Drug induced pemphigus. https://www.ncbi.nlm.nih.gov/books/NBK499864/.

